# Sex-specific lateralization of the effects of exogenously-induced neuroimmune activation in the amygdala on pain-like behaviors

**DOI:** 10.3389/fphar.2026.1883574

**Published:** 2026-07-17

**Authors:** Mariacristina Mazzitelli, Peyton Presto, Sambantham Shanmugam, Nico Antenucci, Garrett Welch, Bovie Liu, Brianna Mendoza, Takaki Kiritoshi, Igor Ponomarev, Volker Neugebauer

**Affiliations:** 1 Department of Pharmacology and Neuroscience, School of Medicine, Texas Tech University Health Sciences Center, Lubbock, TX, United States; 2 Center of Excellence for Translational Neuroscience and Therapeutics, Texas Tech University Health Sciences Center, Lubbock, TX, United States; 3 Garrison Institute on Aging, Texas Tech University Health Sciences Center, Lubbock, TX, United States

**Keywords:** amygdala, behavior, lateralization, neuroimmune system, neuroplasticity, pain, sex differences

## Abstract

**Introduction:**

Chronic pain is a global healthcare issue. Mechanistic insight into pain mechanisms in the brain is needed. Increasing evidence has demonstrated a critical role for neuroimmune signaling factors in the pathogenesis of chronic pain. The amygdala, a bilateral limbic structure, is involved in the emotional-affective dimensions of pain and pain-modulation. There is good evidence for pain-related hemispheric lateralization in the central nucleus of the amygdala (CeA) in different pain models, but pain-related lateralization of neuroimmune signaling in the CeA remains to be determined. This study addressed the question if the well-documented right-hemispheric lateralization in pain conditions also occurs with the exogenous activation of neuroimmune signaling, which would suggest intrinsic differences between right and left CeA as the basis for pain-related hemispheric lateralization.

**Methods:**

To do so, either lipopolysaccharide (LPS), a toll-like receptor 4 (TLR4) agonist, or polyinosinic:polycytidylic acid (PolyI:C), a TLR3 agonist, was injected stereotaxically into the CeA. Mechanosensitivity, emotional-affective responses, and anxiety-like behaviors were evaluated in male and female rats 3 and 7 days post drug administration. Left and right CeA were collected at the end of the experiments for Quantitative Reverse Transcription Polymerase Chain Reaction (qRT-PCR) analysis from female rats.

**Results:**

Our results suggest that both right and left CeA are capable of generating sensory and emotional pain-like behaviors when activated exogenously by immunostimulants at the early stage (day 3) of neuroimmune activation in both sexes. At the later stage (day 7), sex-, treatment- and hemisphere-specific differences emerged with a TLR3-mediated right hemispheric CeA lateralization in females but not in males. qRT-PCR analysis confirmed local neuroimmune activation in both right and left CeA, with treatment-dependent gene expression changes occurring mainly ipsilateral to the injection site.

**Discussion:**

Therefore, exogenous neuroimmune activation is possible in both the left and right CeA and generates pain-like behaviors initially, whereas females, but not males, develop lateralization subsequently, perhaps suggesting resilience developing in the left but not right CeA in terms of output and coupling to pain modulatory systems. Mechanistic insights into the hemispheric lateralization of neuroimmune signaling-related pain modulation in the amygdala may aid the development of therapeutic strategies for chronic pain relief.

## 1 Introduction

Chronic pain is a devastating condition with unmet clinical needs (Stretanski et al., 2025). The amygdala, an almond-shaped bilateral region of the limbic system, is a key structure for emotional processing in the brain, particularly for fear and anxiety. The amygdala attaches emotional significance to nociceptive signals, contributing to the emotional-affective pain responses and pain modulation (Neugebauer, 2020; Thompson and Neugebauer, 2017). Hemispheric asymmetries in various regions of the brain have been studied in human and rodent models of pain. A strong body of evidence from preclinical studies suggests lateralization to the right amygdala in pain, while the left amygdala may exhibit antinociceptive functions (Thompson and Neugebauer, 2017; Kolber et al., 2026; Ji and Neugebauer, 2009). The central nucleus of amygdala (CeA) serves major output functions and receives nociceptive information via the external lateral parabrachial nucleus (PB). A potentiation of the CeA activity and PB transmission of nocicpetive inputs was observed in the right hemisphere regardless of side of injury in inflammatory (formalin) or arthritis pain models (Ji and Neugebauer, 2009; Miyazawa et al., 2018; Sugimura et al., 2016; Han et al., 2005). It is not clear, however, if hemispheric lateralization is only observed in pain conditions or if both sides are able in principle to undergo changes that translate into pain behaviors.

The neuroimmune system is essential for neuronal development and other physiological functions. Alterations of this system have been implicated in peripheral and central pain mechanisms and more recently in the chronification of pain. Within the neuroimmune system the activation of toll-like receptors (TLRs) engages pro-inflammatory processes that enable the release of various cytokines resulting in the maintenance of pain (La et al., 2018; Liu et al., 2022). Strongest evidence for the critical role for the neuroimmune signaling in pain mechanisms still comes from studies at the spinal and peripheral levels. Less is known about the central neuroimmune-mediated pain signaling and the effects of the exogenously induced neuroinflammation in the brain (CeA) on pain processes.

Importantly, sex differences have been described in pain processing in preclinical and clinical settings (Presto et al., 2022). Additionally, the neuroimmune system is significantly sexually dimorphic, and important differences with respect to the neuroimmune-related pain mechanisms have been identified (Presto et al., 2022). At the spinal level, microglia have been implicated in nociceptive responses in male mice, while the adaptive immune system (T-cells) seems to be involved in females (Sorge et al., 2015). Male microglia-mediated pain mechanisms have been confirmed peripherally, but the sex-specific neuroimmune contribution at the supraspinal level remains unclear, particularly with respect to the affective pain circuits. In the nervous system, the activation of TLRs on glial cells (microglia, astrocytes and oligodendrocytes) can modulate neuronal functions involved in nociceptive signaling leading to inflammatory and chronic pain (La et al., 2018; Liu et al., 2022).

This study aims to determine the contribution of the exogenous activation of the neuroimmune response in the left and right CeA to pain-like behaviors. We used local administration of two potent immunostimulants: lipopolysaccharide (LPS), a TLR4 agonist, and polyinosinic:polycytidylic acid (PolyI:C), a TLR3 agonist. The effects of the treatments were evaluated at two different time points, 3 or 7 days after microinjections, in male and female animals.

## 2 Materials and methods

Experimental procedures were approved by the Institutional Animal Care and Use Committee (IACUC; protocol #21026) at Texas Tech University Health Sciences Center and conform to the guidelines of the International Association for the Study of Pain (IASP) and National Institutes of Health (NIH). Male and female wild type rats on Sprague Dawley (SD) background (from our breeding colony) or SD rats purchased from Envigo (Indianapolis, IN) were used in the experimental procedures. All animals, 250–350 g at time of testing, were housed in a temperature-controlled room under a 12 h day/night cycle with unrestricted access to food and water. Rats were randomly assigned to the different experimental groups, and investigators blinded to drug treatments carried out the experimentation.

### 2.1 Drugs

Lipopolysaccharide (LPS; Sigma-Aldrich, St. Louis, MO, catalog #L3024) or polyinosinic:polycytidylic acid (PolyI:C; InvivoGen, San Diego, CA, catalog #tlrl-pic) was used in this study. LPS or PolyI:C was dissolved in ACSF, composed of the following (in mM): 117 NaCl, 4.7 KCl, 1.2 NaH2PO4, 2.5 CaCl2, 1.3 MgCl2, 25 NaHCO3, and 11 glucose. Standard protocols from the manufacturer were used to obtain the final concentration of 1 μg/1 μL stock solution. Aliquots (50 μL) were stored at −80 °C.

### 2.2 Intra-CeA micro-injections

Animals were anesthetized with isoflurane (2%–3%; precision vaporizer, Harvard Apparatus, Holliston, MA, United States) and a small unilateral craniotomy was performed using a stereotaxic apparatus (David Kopf Instruments, Tujunga, CA, United States) as previously described (Mazzitelli et al., 2024). LPS, a TLR4 agonist, or PolyI:C, a TLR3 agonist, was injected into either the left or right CeA to probe the neuroinflammatory state. LPS, PolyI:C (1 μg/μL) or vehicle (ACSF) was injected (1 μL, 10 min) into the left or right CeA with a 5 μL Hamilton syringe using the following coordinates: 2.5 mm caudal to bregma, 4.0–4.3 mm lateral to midline, and 7.3–7.6 mm deep. We did not verify the injection site histologically because all tissue was used for qRT-PCR but we confirmed the microsyringe mark in the brain and the target region. Our previous studies confirmed that drug diffusion did not exceed a radius of 0.5 mm from the injection site based on off-site injections into adjacent striatum (Ji et al., 2013) and differential effects with CeA and BLA injections (Thompson et al., 2015; Ji et al., 2017). Topical antibiotic (Bacitracin) was applied following the craniotomy to prevent infection. Behavioral assays were then performed either 3 or 7 days after the injection.

### 2.3 Behavioral assays

Behavioral assays were performed 3 or 7 days after intra-CeA microinjection of LPS, PolyI:C or vehicle (see 2.2). The following behavioral assays were performed in shielded temperature- and light-controlled rooms. Each rat underwent all behavioral assays in the following order: nocifensive reflex threshold assessment using von Frey test, spontaneous anxiety-like behavior, stimulus-evoked emotional-affective behavior (evoked vocalizations), and nocifensive reflex thresholds using a calibrated forceps. The evoked behavior tests (von Frey, reflexes and vocalizations) were performed on both contra- and ipsi-lateral (to the side of CeA injection) hind paws and were repeated twice in the same animal and then averaged.

#### 2.3.1 Mechanosensitivity

Nocifensive reflex thresholds were assessed using a plantar electronic von Frey anesthesiometer (IITC Life Science, Woodland Hills, CA) or a calibrated forceps connected to a force transducer whose output was displayed in grams on an LCD screen to gradually compress the hind paw with continuously increasing intensities (paw pressure test); the force required for evoking a reflex response was displayed in gram on an LCD screen and recorded (Mazzitelli et al., 2022).

#### 2.3.2 Anxiety-like behaviors

The elevated plus maze (EPM) and open field test (OFT) were used to investigate anxiety-like behaviors as described before (Mazzitelli et al., 2022). Behavioral activity was videotracked for 15 min with EthoVision (Noldus Information Technology, Leesburg, VA, United States). EPM consisted of two open arms and two enclosed arms (10 × 50 cm each) connected by a central area measuring 10 × 10 cm, 60 cm above the floor. At the beginning of each trial, the rat was placed in the center facing one open arm. Open arm choice was calculated as the percentage of total time the rat spent in the open arms in the first 5 min of the session. Locomotor activity was measured as the total distance (cm) the rat traveled during the first 5 min of the assay. OFT consists of a square arena (70 cm × 70 cm) that the animal explores freely. Time spent in the center area of the field (35 cm × 35 cm) and locomotor activity as the total distance traveled were calculated for the first 5 min. Avoidance of the open arms of the EPM or center area of the OFT was interpreted as anxiety-like behavior. Each rat underwent first OFT and then EPM.

#### 2.3.3 Emotional responses

Vocalizations in the audible (20 Hz–16 kHz) and ultrasonic (25 ± 4 kHz) ranges were measured as described before (Mazzitelli et al., 2022; Presto and Neugebauer, 2022). Rats were briefly anesthetized with isoflurane (2%–3%, precision vaporizer) to minimize stress of handling, and placed in a custom-designed recording chamber (U.S. Patent 7,213,538) to ensure a fixed distance from the sound detectors. A microphone connected to a preamplifier was used to record audible vocalizations, and a bat detector connected to a filter and amplifier measured ultrasonic vocalizations (UltraVox four-channel system; Noldus Information Technology). After recovery from the brief anesthesia, vocalizations were evoked by brief (10 s) normally innocuous (300–500 g/6 mm^2^), and noxious (1,000–1,500 g/6 mm^2^) stimuli applied to the contra- or ipsi-lateral hind paws using a calibrated forceps (see “Mechanosensitivity”). Vocalizations were recorded for 1 min and analyzed using Ultravox 2.0 software (Noldus Information Technology). Innocuous stimulation preceded noxious stimulation.

### 2.4 qRT-PCR

At the end of the behavioral sessions (see 2.3), rats were euthanized by decapitation. Brains were rapidly extracted and oxygenated in an ice-cold sucrose-based physiological solution of the following composition (in mM): 87 NaCl, 75 sucrose, 25 glucose, 5 KCl, 21 MgCl_2_, 0.5 CaCl_2_, and 1.25 NaH_2_PO_4_. Two coronal brain slices (1,000 μM) containing the amygdala were prepared using a Vibratome (VT1200S, Leica Biosystems, Nussloch, Germany) as described previously (Mazzitelli et al., 2024; Presto and Neugebauer, 2022; Presto et al., 2023). Both ipsi- or contra-lateral (to the side of injection) amygdala of each rat were dissected for gene expression analysis using quantitative Polymerase Chain Reaction (qPCR). Total RNA was extracted using the MagMAX-96 Total RNA Isolation Kit (Life Technologies, Carlsbad, CA, United States) and quantified on a NanoDrop One spectrophotometer (Thermo Fisher Scientific, Rockford, IL, United States). 1.5 µg total RNA was reverse-transcribed using the Applied BioSystems High-capacity cDNA Reverse Transcription Kit in a 40 µL reaction and diluted at a 1:2 ratio with Nuclease free water. Real time qPCR (qRT-PCR) amplification was performed on a Biorad CFX384 Real time system (Hercules, CA). A 10 µL real time qPCR reaction containing 2 µL of the cDNA, 5 µL of TaqMan™ Fast Advanced Master Mix (Applied Biosystems, Bedford, MA, United States), 20 pmol of the respective primer/probe mix was used to determine the mRNA expression (Table 1). PCR cycling conditions included UNG activation at 50 °C for 2 min, an initial denaturation step at 95 °C for 30s followed by 40 PCR cycles at 95 °C for 5 s and 60 °C for 30 s. The expression of the target gene was determined relative to the geometric mean of the following housekeeping genes: *Actnb*, *Rpl3* and *Rpl29*; these reference genes have been shown to exhibit consistent expression in a rat neuropathic pain model (Wan et al., 2010) and served as reliable internal markers for the analysis of mRNA expression within the CeA tissue of neuropathic rats in our prior studies (Mazzitelli et al., 2024; Presto and Neugebauer, 2022; Presto et al., 2023). Relative expression was determined using the 2^−ΔΔCT^ method with samples normalized to the geometric mean of *Actnb*, *Rpl3* and *Rpl29* (Schmittgen and Livak, 2008). qRT-PCR was performed only in female animals because of the larger effect sizes of the behavioral results that justified the further investigation of molecular mechanisms.

**TABLE 1:** List of genes and their TaqMan probes used.

Gene symbol	Gene name	Accession #	Dye
*Bdnf*	Brain-derived neurotrophic factor	Rn02531967_s1	FAM
*Ccl5*	Chemokine (C-C motif) ligand 5	Rn00579590_m1	FAM
*Cxcl10*	Chemokine (C-X-C motif) ligand 10	Rn01413889_g1	FAM
*Cxcl12*	Chemokine (C-X-C motif) ligand 12	Rn00573260_m1	FAM
*Il23r*	Interleukin 23 receptor	Rn01425151_m1	FAM
*Olig1*	Oligodendrocyte transcription factor 1	Rn00572904_s1	FAM
*Reln*	Reelin	Rn00589609_m1	FAM
*Gfap*	Glial fibrillary acidic protein	Rn01253033_m1	FAM
*Tlr3*	Toll-like receptor 3	Rn01488472_g1	FAM
*Tlr4*	Toll-like receptor 4	Rn01458370_m1	FAM
*ActB*	Beta actin	Rn00667869_m1	VIC
*Rpl3*	Ribosomal protein L3	Rn01505100_g1	VIC
*Rpl29*	Ribosomal protein L29	Rn00820801_g1	VIC

### 2.5 Data and statistical analysis

All averaged values are presented as the means ± SEM. GraphPad Prism 10.0 software (Graph-Pad Software, San Diego, CA) was used for all statistical analyses. Statistical significance was accepted at the level p < 0.05. For behavioral assays, two-way ANOVA with Dunnett’s posthoc tests was used for multiple comparisons. Grubbs’ test was used to identify outliers which were excluded from the statistical analysis of the behavioral assays. For qPCR, the effect of treatment was performed on ΔΔCt values within injection side using one-way ANOVA and when appropriate posthoc comparisons were conducted using Dunnett’s multiple comparisons test to compare each treatment group with the control group. For data visualization, relative expression values are presented as mean ± SEM.

## 3 Results

Behavioral assays were done 3 or 7 days after LPS, PolyI:C, or vehicle micro-injection into the right or left CeA in male and female rats to determine any sex- and hemisphere-specific and time-dependent effects of the exogenous immune activation on pain-like behaviors.

### 3.1 Effects of LPS and PolyI:C on evoked pain-like behaviors in females

#### 3.1.1 3-day time point

**FIGURE 1:**
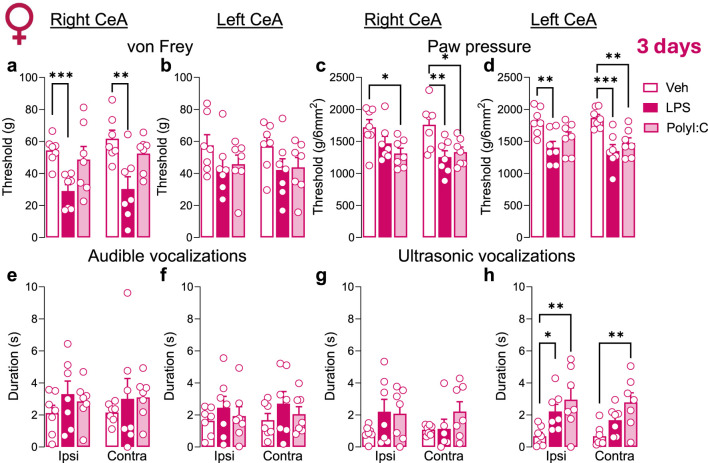
Effects of LPS and PolyI:C delivered into the left or right CeA of female rats on pain-like behaviors 3 days after injection. LPS (1 μg/μL) administered into the right CeA had pronociceptive effects compared to the vehicle (ACSF) treated group in the von Frey test, while PolyI:C (1 μg/μL) showed no effects **(a)**. A non-significant facilitatory trend was observed when either LPS or PolyI:C was injected into the left CeA **(b)**. In the paw pressure test, LPS increased mechanosensitivity of the contra- but not ipsi-lateral paw when injected into the right CeA and had similar pronociceptive effects of both hind paws when delivered into the left CeA **(c,d)**. No effects were observed on the audible vocalizations evoked by the noxious (1,000–1,500 g/6 mm^2^) stimulation of either hind paw regardless of the treatments and sides of injection **(e,f)**. PolyI:C injected into the left, but not right, CeA increased the duration of the ultrasonic vocalizations evoked by the noxious compression of the contra- and ipsi-lateral paws compared to the vehicle treated group **(g,h)**. Similarly, LPS into the left, but not right, CeA increased the ultrasonic vocalizations of the ipsi- but not contra-lateral paw **(g,h)**. Bar histograms show means ± SEM. *, **, ***p< 0.05, 0.01, 0.001 compared to veh, n = 7 per group; Two-way ANOVA with Dunnett’s posthoc tests.

LPS, but not PolyI:C, injection into the right CeA significantly decreased the nocifensive thresholds on the ipsi- and contra-lateral hind paws in the von Frey test compared to the vehicle group (Figure 1a, two-way ANOVA, Contra vs. Ipsi, F_(1, 36)_ = 0.7719, p = 0.3854, Treatment, F _(2, 36)_ = 13.11, p < 0.0001, Interaction, F_(2, 36)_ = 0.1361, p = 0.8732). A similar but non-significant trend was observed for both LPS and PolyI:C applied into the left CeA (Figure 1b, two-way ANOVA, Contra vs. Ipsi, F_(1, 36)_ = 0.0749, p = 0.7859, Treatment, F_(2, 36)_ = 3.056, p = 0.0594, Interaction, F_(2, 36)_ = 0.0068, p = 0.9933). In the paw pressure test, LPS injected into the right CeA decreased the mechanical thresholds of the contra- but not ipsi-lateral paw compared to vehicle injected animals, and similar facilitatory effects were observed on both paws when LPS was injected into the left CeA (Figures 1c,d, two-way ANOVA, (c) Contra vs. Ipsi, F_(1, 36)_ = 0.3607, p = 0.5519, Treatment, F_(2, 36)_ = 9.472, p = 0.0005, Interaction, F_(2, 36)_ = 0.8960, p = 0.4171; (d) Contra vs. Ipsi, F_(1, 36)_ = 0.1985, p = 0.6586, Treatment, F_(2, 36)_ = 15.64, p < 0.0001, Interaction, F_(2, 36)_ = 0.2977, p = 0.7444). PolyI:C administered into the right CeA increased mechanical sensitivity on both hind paws but application into the left CeA had a significant effect only on the ipsi-lateral hind paw (Figures 1c,d). No significant effects of LPS or PolyI:C were observed on the evoked vocalizations in the audible range (Figures 1e,f, two-way ANOVA, (e) Contra vs. Ipsi, F_(1, 36)_ = 3.223, p = 0.9955, Treatment, F_(2, 36)_ = 1.161, p = 0.3245, Interaction, F_(2, 36)_ = 0.07386, p = 0.9289; (f) Contra vs. Ipsi, F_(1, 36)_ = 0.09706, p = 0.7572, Treatment, F_(2, 36)_ = 1.377, p = 0.2652, Interaction, F_(2, 36)_ = 0.01788, p = 0.9823). PolyI:C injected into the left, but not right, CeA significantly increased the ultrasonic vocalizations evoked by the noxious stimulation of the contra- and ipsi-lateral hind paws compared to the vehicle group, while LPS in the left CeA increased the evoked vocalizations of the ipsi-lateral paw only (Figures 1g,h, two-way ANOVA, (g) Contra vs. Ipsi, F_(1, 35)_ = 0.3079, p = 0.5825, Treatment, F_(2, 35)_ = 2.482, p = 0.0982, Interaction, F_(2, 35)_ = 0.8647, p = 0.4300; (h) Contra vs. Ipsi, F_(1, 36)_ = 0.3841, p = 0.5393, Treatment, F_(2, 36)_ = 10.98, p = 0.0002, Interaction, F_(2, 36)_ = 0.1759, p = 0.8394). Similarly, only LPS in the left CeA had significant facilitatory effects on the ultrasonic vocalizations evoked by innocuous stimulation of both paws (Supplementary Figures S1a-d). These data suggest that the exogenous activation of neuroimmune signaling in both the left and right CeA can facilitate pain-related behaviors 3 days after induction in female rats.

#### 3.1.2 7 day-time point

**FIGURE 2:**
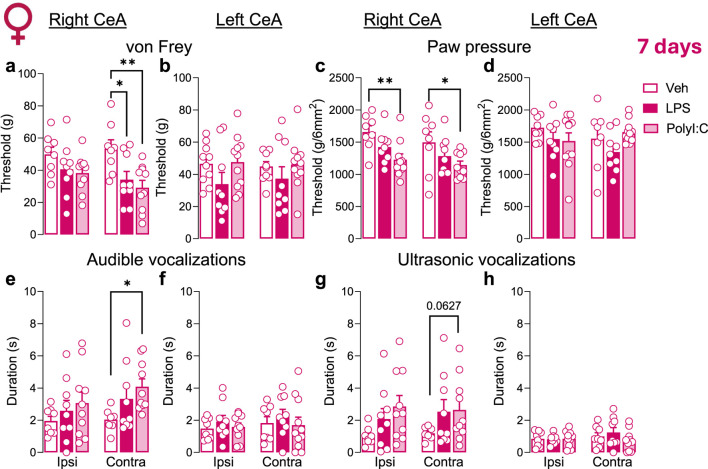
Effects of LPS and PolyI:C delivered into the left or right CeA of female rats on pain-like behaviors 7 days after injection. Both LPS (1 μg/μL) and PolyI:C (1 μg/μL) delivered onto the right, but not left, CeA decreased the mechanical withdrawal thresholds of the contra- but not ipsi-lateral paw compared to the vehicle injected group in the von Frey test **(a,b)**. Similarly, PolyI:C into the right, but not left, CeA had facilitatory effects on both hind paws in the paw pressure test, while LPS had no effects **(c,d)**. PolyI:C injected into the right, but not left, CeA increased the audible vocalizations evoked by the noxious stimulation of the contra-, but not ipsi-, lateral paw, while no effects were observed with LPS in either hemisphere **(e,f)**. No significant effects were detected on the ultrasonic vocalizations regardless of the treatments and hemispheres **(g,h)**. Bar histograms show means ± SEM. *, **p< 0.05, 0.01, compared to veh, n = 8–10 per group; Two-way ANOVA with Dunnett’s posthoc tests.

LPS and PolyI:C injected into the right, but not left, CeA significantly decreased the nocifensive thresholds on the contra-lateral paw and decreased the ipsi-lateral withdrawal thresholds in a non-significant fashion in the von Frey test compared to the vehicle treated group (Figures 2a,b, two-way ANOVA, (a) Contra vs. Ipsi, F_(1, 48)_ = 1.027, p = 0.3161, Treatment, F_(2, 48)_ = 7.329, p = 0.0017, Interaction, F_(2, 48)_ = 0.9038, p = 0.4118; (b) Contra vs. Ipsi, F_(1, 54)_ = 0.0007, p = 0.9796, Treatment, F_(2, 54)_ = 2.609, p = 0.0829, Interaction, F_(2, 54)_ = 0.1356, p = 0.8735). PolyI:C, but not LPS, administered into the right, but not left, CeA increased mechanical sensitivity on both paws in the paw pressure test (Figures 2c,d, two-way ANOVA, (c) Contra vs. Ipsi, F_(1, 48)_ = 2.449, p = 0.1242, Treatment, F_(2, 48)_ = 7.590, p = 0.0014, Interaction, F_(2, 48)_ = 0.0833, p = 0.9202; (d) Contra vs. Ipsi, F_(1, 50)_ = 0.7737, p = 0.3833, Treatment, F_(2, 50)_ = 1.624, p = 0.2073, Interaction, F_(2, 50)_ = 1.676, p = 0.1976). Similarly, PolyI:C into the right, but not left, CeA increased the audible vocalizations evoked by noxious stimulation of the contra-lateral paw, (Figures 2e,f, two-way ANOVA, (e) Contra vs. Ipsi, F_(1, 47)_ = 1.641, p = 0.2065, Treatment, F_(2, 47)_ = 3.534, p = 0.0372, Interaction, F_(2, 47)_ = 0.3141, p = 0.7319; (f) Contra vs. Ipsi, F_(1, 49)_ = 0.6992, p = 0.4071, Treatment, F_(2, 49)_ = 0.8448, p = 0.4358, Interaction, F_(2, 49)_ = 0.0602, p = 0.9417). LPS had no effects on the evoked audible vocalizations (Figures 2e,f). No facilitatory effects were observed on the duration of the noxious stimulus-evoked ultrasonic vocalizations (Figures 2g,h, two-way ANOVA, (g) Contra vs. Ipsi, F_(1, 47)_ = 0.0819, p = 0.7760, Treatment, F_(2, 47)_ = 3.758, p = 0.0306, Interaction, F_(2, 47)_ = 0.1661, p = 0.8474; (h) Contra vs. Ipsi, F_(1, 47)_ = 0.8215, p = 0.3694, Treatment, F_(2, 47)_ = 0.6317, p = 0.5361, Interaction, F_(2, 47)_ = 0.8280, p = 0.4432) and on the vocalizations evoked by innocuous stimulation of both paws regardless of the treatments and hemispheres (Supplementary Figures S1e,h). These findings suggest that the facilitatory effects of PolyI:C on sensory and affective responses and the nocifensive effects of LPS last 7 days after injection and develop a right-hemispheric lateralization of CeA neuroimmune signaling linked to pain-like behaviors in female animals.

Overall, our data suggest that exogenously-induced neuroimmune system activation in the CeA can generate pain-like behaviors in naïve female rats. Despite some variability, these effects were not lateralized at the 3 day-time point but developed right-hemispheric lateralization in the CeA for PolyI:C, and to some extent for LPS, 7 days after injections in the female groups, supporting the idea that predominantly TLR3-mediated lateralization develops over time in the right CeA.

### 3.2 Effects of LPS and PolyI:C on evoked pain-like behaviors in males

#### 3.2.1 3-day time point

**FIGURE 3:**
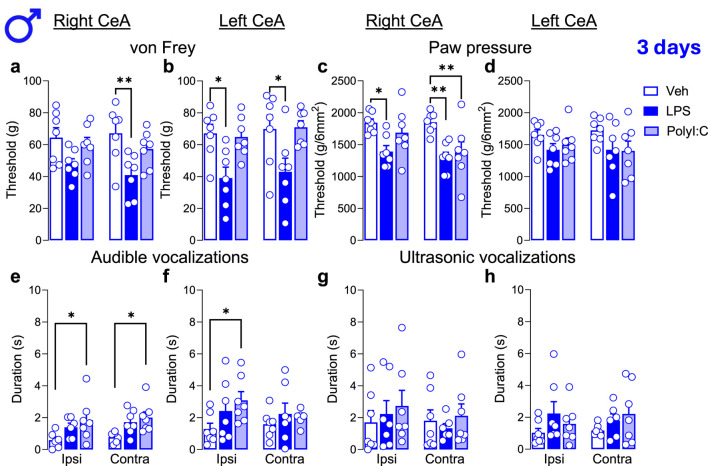
Effects of LPS and PolyI:C delivered into the left or right CeA of male rats on pain-like behaviors 3 days after injection. LPS (1 μg/μL), but not PolyI:C (1 μg/μL), decreased the mechanical withdrawal thresholds of the contra-lateral paw when injected in the right CeA, and of both hind paws when injected in the left CeA compared to the vehicle (ACSF) treated group in the von Frey test **(a,b)**. In the right, but not left, CeA LPS induced hypersensitivity in the contra- and ipsi-lateral paws, while PolyI:C had facilitatory effects only on the contra-lateral hind paw **(c,d)**. PolyI:C, but not LPS, increased the duration of the audible vocalizations evoked by the noxious (1,000–1,500 g/6 mm^2^) stimulation of both hind paws when injected in the right CeA and only of the ipsi-lateral paw when injected into the left CeA **(e,f)**. No effects were detected on the vocalizations measured in the ultrasonic range regardless of the treatments and hemispheres **(g,h)**. Bar histograms show means ± SEM. *, **p <0.05, 0.01, compared to veh, n = 7 per group; Two-way ANOVA with Dunnett’s posthoc tests.

Rats showed decreased nocifensive thresholds on the contra-lateral paw in the von Frey test when LPS, but not PolyI:C, was injected into the right CeA, and hypersensitivity was observed on both hind paws when LPS, but not PolyI:C, was administered into the left CeA (Figures 3a,b, two-way ANOVA, (a) Contra vs. Ipsi, F_(1, 36)_ = 0.2819, p = 0.5987, Treatment, F_(2, 36)_ = 8.720, p = 0.0008, Interaction, F_(2, 36)_ = 0.4244, p = 0.6574; (b) Contra vs. Ipsi, F_(1, 36)_ = 0.5975, p = 0.4446, Treatment, F_(2, 36)_ = 11.00, p = 0.0002, Interaction, F_(2, 36)_ = 0.0321, p = 0.9684) compared to the vehicle group. LPS in the right, but not left, CeA increased the mechanosensitivity on both hind paws in the paw pressure test, while PolyI:C in the right, but not left, CeA had significant facilitatory effects on the contra-lateral paw only (Figures 3c,d, two-way ANOVA, (c) Contra vs. Ipsi, F_(1, 36)_ = 2.204, p = 0.1464, Treatment, F _(2, 36)_ = 10.71, p = 0.0002, Interaction, F_(2, 36)_ = 1.080, p = 0.3504; (d) Contra vs. Ipsi, F_(1, 36)_ = 0.0080, p = 0.9293, Treatment, F_(2, 36)_ = 3.167, p = 0.0541, Interaction, F_(2, 36)_ = 0.2657, p = 0.7681). PolyI:C injected into the right CeA enhanced the duration of audible vocalizations evoked by the noxious stimulation of both hind paws, while PolyI:C administration into the left CeA only affected the vocalizations to stimulation of the ipsi-lateral paw (Figures 3e,f, two-way ANOVA, (e) Contra vs. Ipsi, F_(1, 33)_ = 0.6576, p = 0.4232, Treatment, F_(2, 33)_ = 7.269, p = 0.0024, Interaction, F_(2, 33)_ = 0.0320, p = 0.9685; (f) Contra vs. Ipsi, F_(1, 35)_ = 0.5895, p = 0.4478, Treatment, F_(2, 35)_ = 2.559, p = 0.0918, Interaction, F_(2, 35)_ = 0.9078, p = 0.4127), and had no significant effects on the ultrasonic vocalizations (Figures 3g,h, two-way ANOVA, (g) Contra vs. Ipsi, F_(1, 35)_ = 0.5847, p = 0.4496, Treatment, F_(2, 35)_ = 0.5104, p = 0.6047, Interaction, F_(2, 35)_ = 0.2276, p = 0.7976; (h) Contra vs. Ipsi, F_(1, 35)_ = 0.0670, p = 0.7972, Treatment, F_(2, 35)_ = 1.961, p = 0.1558, F_(2, 35)_ = 0.5498, p = 0.5820). LPS had no effects on the evoked vocalizations evoked by the noxious paw stimulation in the audible or ultrasonic ranges regardless of the treatments or hemispheres (Figures 3e–h). Additionally, audible and ultrasonic vocalizations evoked by the innocuous stimulation of the hind paws were not affected by any treatment except for LPS in the left CeA that enhanced the ultrasonic vocalizations evoked by the stimulation of the ipsi-lateral paw (Supplementary Figures S1i,l). These data suggest that activation of TLR3 and TLR4 in both the left and right CeA can drive pain-like responses 3 days after LPS or PolyI:C injection in male rats, but with a tendency for TLR4 couple to nocifensive and TLR3 to affective responses in males.

#### 3.2.2 7-day time point

**FIGURE 4:**
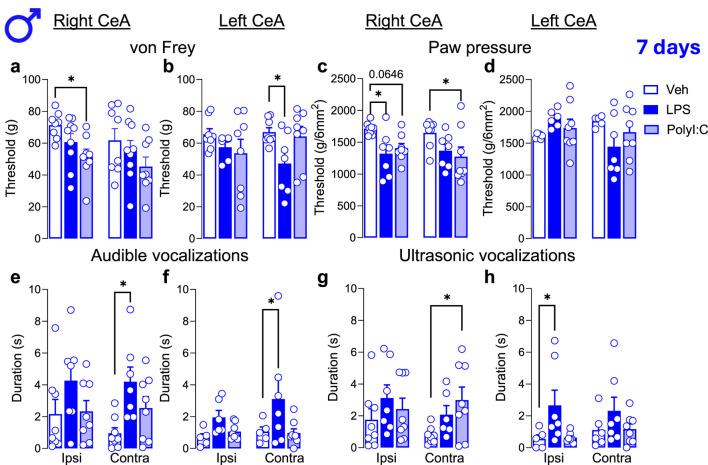
Effects of LPS and PolyI:C delivered into the left or right CeA of male rats on pain-like behaviors 7 days after injection. PolyI:C (1 μg/μL) delivered into the right CeA decreased the mechanical thresholds of the ipsi- but not contra-lateral paw in the von Frey test, while LPS (1 μg/μL) into the right CeA had no effects **(a)**. LPS into the left CeA had facilitatory effects on the contra- but not ipsi-lateral paw, while no effects were observed with PolyI:C **(b)**. In the paw pressure test, LPS into the right CeA increased mechanosensitivity in the ipsi- but not contra-lateral paw. PolyI:C into the right CeA induced hypersensitivity in the contra-lateral paw and a non-significant facilitatory trend was observed in the ipsi-lateral paw **(c)**. Neither treatment had effect on the paw pressure test when injected into the left CeA **(d)**. LPS, but not PolyI:C, into right and left CeA increased the duration of the audible vocalizations evoked by the noxious stimulation of the contra- but not ipsi-lateral paw **(e,f)**. PolyI:C into the right CeA increased the ultrasonic vocalizations evoked by the noxious stimulation of the contra- but not ipsi-lateral paw, while LPS into the right CeA had no effects **(g)**. LPS injected into the left CeA enhanced the ultrasonic vocalizations elicited by the noxious stimulation of the ipsi- but not contra-lateral paw, while PolyI:C into the left CeA did not show effects **(h)**. Bar histograms show means ± SEM. *, p <0.05, compared to veh, n = 8-9 per group; Two-way ANOVA with Dunnett’s posthoc tests.

PolyI:C in the right, but not left, CeA decreased the nocifensive thresholds on the ipsi- but not contra-lateral hind paw in the von Frey test, compared to the vehicle treated group (Figure 4a, two-way ANOVA, Contra vs. Ipsi, F_(1, 44)_ = 2.261, p = 0.1398, Treatment, F_(2, 44)_ = 4.996, p = 0.0111, Interaction, F_(2, 44)_ = 0.0824, p = 0.9211), while LPS in the left, but not right, CeA increased mechanosensitivity of the contra- but not ipsi-lateral hind paw (Figure 4b, two-way ANOVA, Contra vs. Ipsi, F_(1, 39)_ = 0.0121, p = 0.9131, Treatment, F_(2, 39)_ = 2.689, p = 0.0806, Interaction, F_(2, 39)_ = 1.490, p = 0.2380). In the paw pressure test, PolyI:C in the right CeA significantly decreased the mechanical thresholds on the contra-lateral paw and had a non-significant effect on mechanosensitivity of the ipsi-lateral hind paw (Figure 4c, two-way ANOVA, Contra vs. Ipsi, F_(1, 40)_ = 0.3776, p = 0.5424, Treatment, F_(2, 40)_ = 7.561, p = 0.0016, Interaction, F_(2, 40)_ = 0.3890, p = 0.6803). LPS injected into the right CeA increased mechanosensitivity only on the ipsi-lateral paw (Figure 4c). No treatment in the left CeA induced facilitatory effects in the paw pressure test (Figure 4d, two-way ANOVA, Contra vs. Ipsi, F_(1, 34)_ = 0.6841, p = 0.4140, Treatment, F_(2, 34)_ = 0.1014, p = 0.9039, Interaction, F_(2, 34)_ = 3.127, p = 0.0567). LPS, but not PolyI:C, in the left and right CeA increased the audible vocalizations evoked by the noxious stimulation of the contra- but not ipsi-lateral paw (Figures 4e,f, two-way ANOVA, (e) Contra vs. Ipsi, F_(1, 40)_ = 0.3071, p = 0.5825, Treatment, F_(2, 40)_ = 5.738, p = 0.0064, Interaction, F_(2, 40)_ = 0.4650, p = 0.6315; (f) Contra vs. Ipsi, F_(1, 33)_ = 0.9279, p = 0.3424, Treatment, F_(2, 33)_ = 4.635, p = 0.0168, Interaction, F_(2, 33)_ = 0.6102, p = 0.5492). PolyI:C in the right, but not left, CeA significantly increased the ultrasonic vocalizations evoked by the noxious stimulation of the contra- but not ipsi-lateral hind paw, while LPS injected into the left, but not right, CeA resulted in a significant increase of the duration of the ultrasonic vocalizations evoked by stimulation of the ipsi- but not contra-lateral hind paw (Figures 4g,h, two-way ANOVA, (g) Contra vs. Ipsi, F_(1, 39)_ = 0.7980, p = 0.3772, Treatment, F_(2, 39)_ = 3.022, p = 0.0602, Interaction, F_(2, 39)_ = 0.9750, p = 0.3862; (h) Contra vs. Ipsi, F_(1, 34)_ = 0.2431, p = 0.6252, Treatment, F_(2, 34)_ = 4.916, p = 0.0133, Interaction, F_(2, 34)_ = 0.3697, p = 0.6937). LPS in the right and left CeA had facilitatory effects on the audible vocalizations evoked by the innocuous stimulation of the ipsi- but not contra-lateral hind paws (Supplementary Figures S1m,n), while no significant changes were observed on the ultrasonic vocalizations evoked by the innocuous compression of the hind paws regardless of the treatments and hemispheres (Supplementary Figures S1o,p). These results suggest that the exogenous activation of the neuroimmune system in both the right and left CeA can result in pain-like behaviors 7 days after LPS or PolyI:C treatment in male animals, suggesting that no consistent right-hemispheric lateralization emerged in males, although TLR4-mediated facilitation of affective responses seemed to show a delayed development.

These data point to facilitatory effects of both TLR3 and TLR4 stimulation on pain-like behaviors in naïve male rats. Overall, there was no clear pattern of differences between treatments or hemispheres at the early or late time point, suggesting that both left and right CeA are capable of driving pain-like behaviors.

### 3.3 Effects of LPS and PolyI:C on anxiety-like behaviors in female and male rats

#### 3.3.1 3-day time point

**FIGURE 5:**
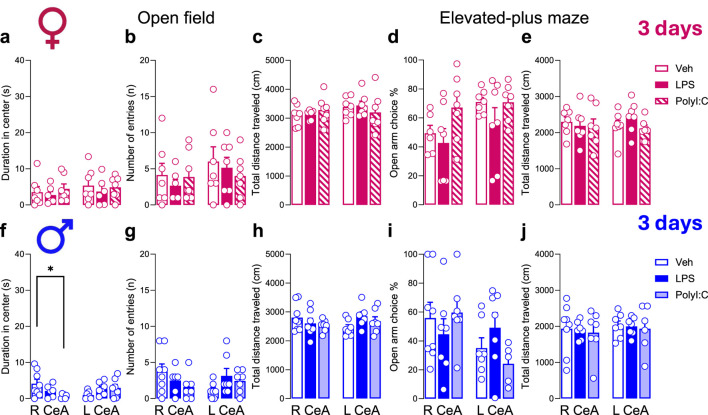
Effects of LPS and PolyI:C delivered into the left or right CeA of male and female rats on anxiety-like behaviors 3 days after injection. In female rats, injection of LPS (1 μg/μL) or PolyI:C (1 μg/μL) in right or left CeA there were no effects on anxiety-like behaviors measured as duration **(a)** and number of entries **(b)** in the center of the OFT and open arm choice **(d)** in the EPM and on locomotor activity (OFT, **(c)**; EPM, **(e)** compared to the vehicle (ACSF) treated group. In male rats, PolyI:C microinjection into the right, but not left, CeA induced a significant decrease in the time spent in the center of the OFT **(f)**. No further effects of PolyI:C were determined in the anxiety-like behaviors and no changes were detected with LPS treatment **(f,g,i)**; no significant changes were detected on the locomotor activity (OFT, **(h)**; EPM, **(j)**). Bar histograms show means ± SEM. *, p < 0.05, compared to veh, n = 7-8 per group; Two-way ANOVA with Dunnett’s posthoc tests.

LPS or PolyI:C injection into the left or right CeA showed no effects on anxiety-like behaviors in the OFT and EPM compared to the vehicle group in female animals (Figures 5a,b,d, two-way ANOVA, (a) Injection side, F_(1, 34)_ = 0.8402, p = 0.3658, Treatment, F_(2, 34)_ = 0.5612, p = 0.5757, Interaction, F_(2, 34)_ = 0.1257, p = 0.8823; (b) Injection side, F_(1, 35)_ = 1.531, p = 0.2242, Treatment, F_(2, 35)_ = 0.4132, p = 0.6647, Interaction, F_(2, 35)_ = 0.3353, p = 0.7174; (d) Injection side, F_(1, 35)_ = 4.153, p = 0.0492, Treatment, F_(2, 35)_ = 3.228, p = 0.0517, Interaction, F_(2, 35)_ = 0.6562, p = 0.5251) or locomotor activity (Figures 5c,e, two-way ANOVA, (c) Injection side, F_(1, 35)_ = 1.622, p = 0.2112, Treatment, F_(2, 35)_ = 0.0335, p = 0.9671, Interaction, F_(2, 35)_ = 0.9135, p = 0.4105; (e) Injection side, F_(1, 36)_ = 0.0530, p = 0.8192, Treatment, F_(2, 36)_ = 0.5684, p = 0.5715, Interaction, F_(2, 36)_ = 0.7141, p = 0.4964). In males, 3 days post treatment a significant increase in anxiety-like behavior, measured as time spent in the center, was observed in the OFT when PolyI:C was injected into the right, but not left, CeA (Figure 5f, two-way ANOVA, Injection side, F_(1, 35)_ = 0.0070, p = 0.9335, Treatment, F_(2, 35)_ = 0.4873, p = 0.6184, Interaction, F_(2, 35)_ = 5.809, p = 0.0066), while no changes were observed in the OFT number of entries in the arena center or EPM (Figures 5g,i, two-way ANOVA, (g) Injection side, F_(1, 36)_ = 0.3193, p = 0.5756, Treatment, F_(2, 36)_ = 0.5126, p = 0.6032, Interaction, F_(2, 36)_ = 3.058, p = 0.0593; (i) Injection side, F_(1, 35)_ = 4.714, p = 0.0368, Treatment, F_(2, 35)_ = 0.1290, p = 0.8794, Interaction, F_(2, 35)_ = 2.115, p = 0.1358) and in locomotor activity (Figures 5h,j, two-way ANOVA, (h) Injection side, F_(1, 37)_ = 0.0246, p = 0.8762, Treatment, F_(2, 37)_ = 0.4414, p = 0.6465, Interaction, F_(2, 37)_ = 2.604, p = 0.0875; (j) Injection side, F_(1, 36)_ = 0.4147, p = 0.5237, Treatment, F_(2, 36)_ = 0.1332, p = 0.8757, Interaction, F_(2, 36)_ = 0.0025, p = 0.9975) regardless of the treatments or side of microinjection.

#### 3.3.2 7-day time point

**FIGURE 6:**
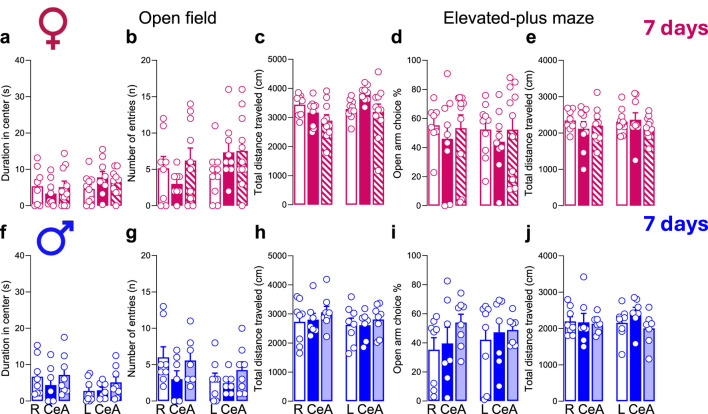
Effects of LPS and PolyI:C delivered into the left or right CeA of male and female rats on anxiety-like behaviors 7 days after injection. No significant effects were detected after LPS (1 μg/μL) or PolyI:C (1 μg/μL) injection regardless of the treatments and hemispheres on anxiety-like behaviors (OFT, (**a,b,f,g)**; EPM, **(d,i)** nor locomotor activity (OFT, **(c,h)**; EPM, **(e,j)** in female or male rats. Bar histograms show means ± SEM, n = 7-9 per group.

No significant changes were observed in avoidance behavior of female rats in the OFT or EPM tests regardless of the treatments or hemispheres (Figures 6a,b,d, two-way ANOVA, (a) Injection side, F _(1, 50)_ = 2.075, p = 0.1560, Treatment, F_(2, 50)_ = 0.1649, p = 0.8484, Interaction, F_(2, 50)_ = 1.597, p = 0.2127; (b) Injection side, F_(1, 50)_ = 1.996, p = 0.1639, Treatment, F_(2, 50)_ = 1.406, p = 0.2548, Interaction, F_(2, 50)_ = 1.604, p = 0.2112; (d) Injection side, F_(1, 51)_ = 0.08732, p = 0.7688, Treatment, F_(2, 51)_ = 0.6591, p = 0.5216, Interaction, F_(2, 51)_ = 0.006174, p = 0.9938). No significant changes in the locomotor activity were detected in the OFT or EPM when LPS or PolyI:C were injected into the left or right, CeA (Figures 6c,e, two-way ANOVA, (c) Injection side, F_(1, 51)_ = 3.269, p = 0.0765, Treatment, F_(2, 51)_ = 3.0467, p = 0.0388, Interaction, F_(2, 51)_ = 2.345, p = 0.1061; (e) Injection side, F_(1, 50)_ = 0.2521, p = 0.6178, Treatment, F_(2, 50)_ = 0.5179, p = 0.5989, Interaction, F_(2, 50)_ = 0.6024, p = 0.5514). No significant effects of LPS or PolyI:C in either right or left CeA were found on anxiety-like behaviors or locomotion in males (Figures 6f–j, two-way ANOVA, (f) Injection side, F_(1, 40)_ = 3.519, p = 0.0680, Treatment, F_(2, 40)_ = 1.155, p = 0.3255, Interaction, F_(2, 40)_ = 0.3224, p = 0.7263; (g) Injection side, F_(1, 39)_ = 3.287, p = 0.0775, Treatment, F_(2, 39)_ = 2.016, p = 0.1468, Interaction, F_(2, 39)_ = 0.6873, p = 0.5089; (h) Injection side, F_(1, 40)_ = 1.035, p = 0.3151, Treatment, F_(2, 40)_ = 0.8636, p = 0.4293, Interaction, F_(2, 40)_ = 0.0590, p = 0.9428; (i) Injection side, F_(1, 39)_ = 0.2134, p = 0.6467, Treatment, F_(2, 39)_ = 1.242, p = 0.2999, Interaction, F_(2, 39)_ = 0.3735, p = 0.6907; (j) Injection side, F_(1, 39)_ = 0.0489, p = 0.8262, Treatment, F_(2, 39)_ = 0.9864, p = 0.3820, Interaction, F_(2, 39)_ = 0.7312, p = 0.4878).

The data suggest that the exogenous activation of neuroimmune signaling in the left or right CeA of female animals does not affect anxiety-like behaviors 3 or 7 days post-treatment. In males, the activation of TLR3 in the right but not left CeA promoted anxiogenic behaviors at the 3-day but not 7-day time point.

### 3.4 LPS or PolyIC- induced gene expression changes in right and left CeA in female rats

To determine if the development of lateralization in females was due to resilience of the left CeA to neuroimmune activation at the molecular level and/or to uncoupling from pain modulatory systems, qPCR analysis was performed on CeA tissue ipsi- or contra-lateral to the side of injection of LPS and PolyI:C in female animals Although, tissues samples were taken from all animals, we elected to focus mRNA expression analysis on female animals only, because of the pronounced lateralization effects in female rather than male animals (Figures 1–4).

#### 3.4.1 3-day time point

**FIGURE 7:**
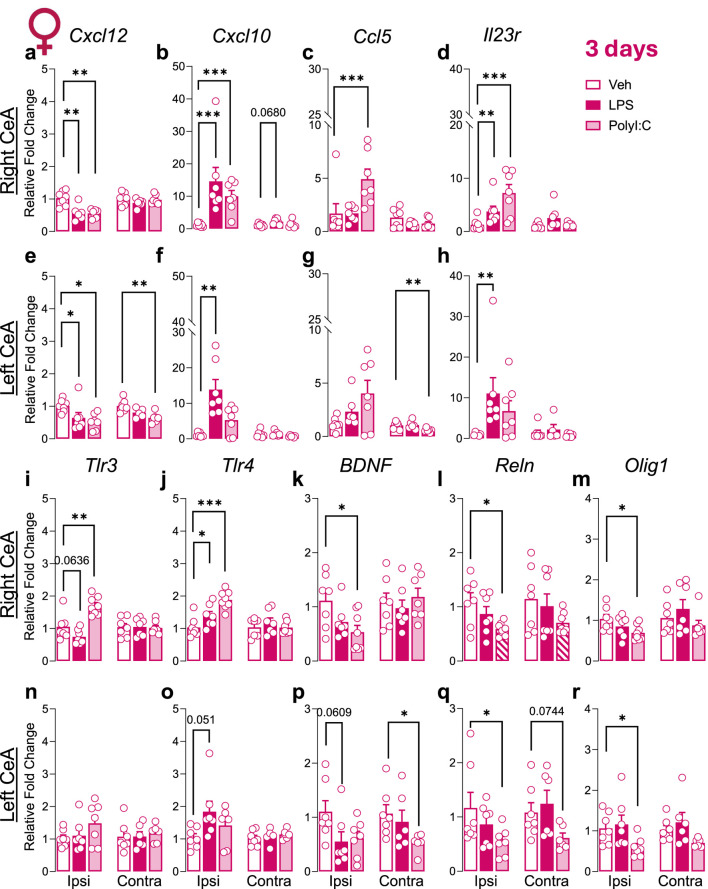
Gene expression changes 3 days after LPS and PolyI:C injection into the left or right CeA of female rats. LPS (1 μg/μL) and PolyI:C (1 μg/μL) injection into the right CeA induced changes in the relative mRNA expression of *Cxcl12* **(a)**, *Cxcl10* **(b)**, *Il23r* **(d)**, *Tlr3* **(i)** and *Tlr4* **(j)** in ipsi-, but not contra-, lateral side. PolyI:C, but not LPS, into the right CeA resulted in significant mRNA expression changes of *Ccl5* **(c)**, *BDNF* **(k)**, *Reln* **(l)** and *Olig1* **(m)** in ipsi-, but not contra-, lateral side. LPS into the left CeA promoted changes in *Cxcl12* **(e)**, *Cxcl10* **(f)**, *Il23r* **(h)** and *Tlr4* **(o)** ipsilaterally to the injection. Left CeA injected PolyI:C down-regulated *Cxcl12* **(e)**, *Reln* **(q)** and *Olig1* **(r)** on both sides, and *Ccl5* **(g)** and BDNF **(p)** only in the contra-lateral side. No significant changes were observed in *Tlr3* **(n)** (left injection), Bar histograms show means ± SEM. *, **, ***p< 0.05, 0.01, 0.001 compared to veh, n = 7 per group; One-way ANOVA with Dunnett’s posthoc tests.

After LPS or PolyI:C injection into the right CeA, significant changes in the expression of *Cxcl12* and *Cxcl10* were observed in the ipsi- but not contra-lateral CeA, while only PolyI:C administration induced significant changes in *Ccl5* and *Il23r* mRNA levels (Figures 7a–d, one-way ANOVA, (a) Ipsi, F_(2, 18)_ = 11.17, p = 0.0007, Contra, F_(2, 18)_ = 1.388, p = 0.2750; (b) Ipsi, F_(2, 18)_ = 31.22, p < 0.0001, Contra, F_(2, 18)_ = 3.766, p = 0.0430; (c) Ipsi, F_(2, 18)_ = 10.78, p = 0.0008, Contra, F_(2, 18)_ = 1.139, p = 0.3420; (d) Ipsi, F_(2, 18)_ = 16.57, p < 0.0001, Contra, F_(2, 17)_ = 2.865, p = 0.0846). LPS injected into the left CeA resulted in significant changes of *Cxcl12*, *Cxcl10* and *Il23r* in the ipsi- but not contra-lateral CeA, while PolyI:C significantly downregulated *Cxcl12* expression in both sides and *Ccl5* expression on the contra-lateral CeA only (Figures 7e–h, one-way ANOVA, (e) Ipsi, F_(2, 18)_ = 5.400, p = 0.0145, Contra, F_(2, 16)_ = 9.001, p = 0.0024; (f) Ipsi, F_(2, 18)_ = 8.191, p = 0.0030, Contra, F_(2, 16)_ = 2.221, p = 0.1408; (g) Ipsi, F_(2, 18)_ = 0.6860, p = 0.5163, Contra, F_(2, 16)_ = 6.850, p = 0.0071; (h) Ipsi, F_(2, 17)_ = 6.308, p = 0.0089, Contra, F_(2, 15)_ = 1.087, p = 0.3624). PolyI:C injection into the right CeA induced significant changes of *Tlr3*, *Tlr4*, *BDNF*, *Reln* and *Olig1* mRNA expression in the ipsi- but not contra-lateral CeA, while LPS into the right CeA resulted in an upregulation of *Tlr4* levels and a non-significant downregulation of *Tlr3* in the ipsi-lateral CeA (Figures 7i–m, one-way ANOVA, (i) Ipsi, F_(2, 18)_ = 17.95, p < 0.0001, Contra, F_(2, 18)_ = 0.001327, p = 0.9868; (j) Ipsi, F_(2, 18)_ = 15.50, p = 0.0001, Contra, F_(2, 18)_ = 0.4794, p = 0.6268; (k) Ipsi, F_(2, 18)_ = 5.866, p = 0.0109, Contra, F_(2, 18)_ = 0.4497, p = 0.6448; (L) Ipsi, F_(2, 18)_ = 3.122, p = 0.0685, Contra, F_(2, 18)_ = 0.9779, p = 0.3952; (m) Ipsi, F_(2, 18)_ = 4.223, p = 0.0313, Contra, F_(2, 18)_ = 1.245, p = 0.3117). LPS administered into the left CeA resulted in a non-significant change of *Tlr4* and *BDNF* levels in the ipsi-, but not contra-lateral CeA (Figures 7n–r). PolyI:C in the left CeA significantly downregulated *Reln* and *Olig1* expression in the ipsi-lateral CeA; it also decreased *BDNF* expression levels and had non-significant effects on *Reln* and *Olig1* in the contra-lateral tissue (Figures 7n–r, one-way ANOVA, (n) Ipsi, F_(2, 18)_ = 1.259, p = 0.3078, Contra, F_(2, 16)_ = 0.2963, p = 0.7475; (o) Ipsi, F_(2, 18)_ = 2.879, p = 0.0823, Contra, F_(2, 16)_ = 0.6383, p = 0.5411; (p) Ipsi, F_(2, 18)_ = 3.033, p = 0.0732, Contra, F_(2, 16)_ = 4.042, p = 0.0379; (q) Ipsi, F_(2, 18)_ = 3.325, p = 0.0591, Contra, F_(2, 16)_ = 3.855, p = 0.0430; (r) Ipsi, F_(2, 18)_ = 5.112, p = 0.0175, Contra, F_(2, 16)_ = 3.989, p = 0.0393). These data suggest that it is possible to induce in the left and right CeA molecular changes of pro-inflammatory factors as well as other critical regulators involved with neuroimmune system and neuronal plasticity.

#### 3.4.2 7-day time point

**FIGURE 8:**
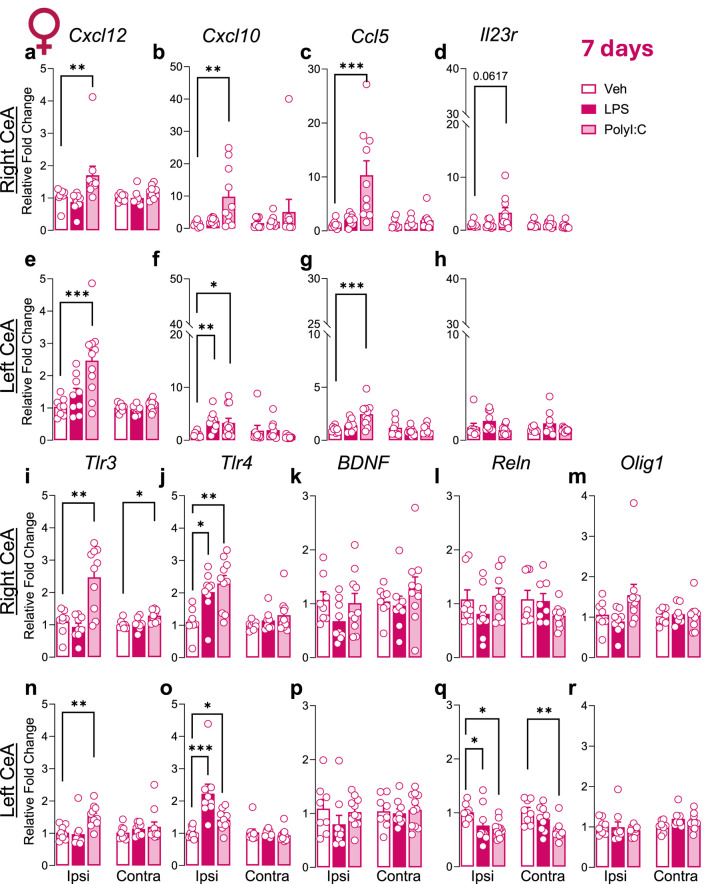
Gene expression changes 7 days after LPS and PolyI:C injection into the left or right CeA of female rats. PolyI:C (1 μg/μL), but not LPS(1 μg/μL), injected into the right CeA up-regulated *Cxcl12* **(a)**, *Cxcl10* **(b)**, *Ccl5* **(c)** and *Il23r* **(d)** in the ipsi-, but not contra-, lateral side, and *Tlr3* **(i)** on both sides. Both LPS and PolyI:C into the right CeA increased mRNA expression of *Tlr4* **(j)** ipsilaterally to the injection. PolyI:C delivered into the left CeA determined changes in the *Cxcl12* **(e)**, *Ccl5* **(g)**, *Tlr3* (**n**) and *Tlr3* **(n)** expression levels in the ipsi-, but not contra-, lateral side. Both LPS and PolyI:C into the left CeA induced changes in *Cxcl10* **(f)**, *Tlr4* **(o)** and *Reln* **(q)** ipsilaterally to the injection. No significant changes were observed in *Il23r* **(h)** (left injection), *BDNF* (right **(k)** or left **(p)** injection), *Reln* **(l)** (right injection) or *Olig1* (right **(m)** or left **(r)** injection). Bar histograms show means ± SEM. *, **, ***p< 0.05, 0.01, 0.001 compared to veh, n = 8–10 per group; One-way ANOVA with Dunnett’s posthoc tests.

PolyI:C, but not LPS, delivered into the right CeA significantly upregulated *Cxcl12*, *Cxcl10* and *Ccl5* in the ipsi-lateral tissue*,* while a non-significant increase of *Il23r* expression was observed at the same time point (Figures 8a–d, one-way ANOVA, (a) Ipsi, F_(2, 24)_ = 6.982, p = 0.0041, Contra, F_(2, 23)=_3.347, p = 0.0530; (b) Ipsi, F_(2, 24)_ = 6.829, p = 0.0045, Contra, F_(2, 23)_ = 0.2830, p = 0.7561; (c) Ipsi, F_(2, 24)_ = 15.57, p < 0.0001, Contra, F_(2, 23)_ = 1.293, p = 0.2936; (d) Ipsi, F_(2, 23)_ = 4.003, p = 0.0322, Contra, F_(2, 21)_ = 1.643, p = 0.2173). Similarly, PolyI:C injected into the left CeA resulted in significant increase of *Cxcl12*, *Cxcl10* and *Ccl5* mRNA levels in the ipsi- but not contra-lateral CeA, while LPS had significant effects on *Cxcl10* levels only (Figures 8e–h, one-way ANOVA, (e) Ipsi, F_(2, 25)_ = 8.893, p = 0.0012, Contra, F_(2, 25)_ = 2.598, p = 0.0944; (f) Ipsi, F_(2, 25)_ = 6.193, p = 0.0065, Contra, F_(2, 25)_ = 3.430, p = 0.0483; (g) Ipsi, F_(2, 25)_ = 8.893, p = 0.0012, Contra, F_(2, 25)_ = 0.5930, p = 0.5602; (h) Ipsi, F_(2, 25)_ = 4.073, p = 0.0300, Contra, F_(2, 23)_ = 1.656, p = 0.2129). PolyI:C and LPS in the right CeA induced an ipsi-lateral upregulation of *Tlr4*, while changes in *Tlr3* mRNA expression on both CeA were induced only by the PolyI:Cinjection into the right CeA (Figures 8i–m, one-way ANOVA, (i) Ipsi, F_(2, 24)_ = 10.28, p = 0.0006, Contra, F_(2, 23)_ = 6.406, p = 0.0061; (j) Ipsi, F_(2, 24)_ = 6.295, p = 0.0063, Contra, F_(2, 23)_ = 1.407, p = 0.2653; (k) Ipsi, F_(2, 24)_ = 2.334, p = 0.1185, Contra, F_(2, 23)_ = 0.2541, p = 0.7778; (L) Ipsi, F_(2, 24)_ = 2.552, p = 0.0989, Contra, F_(2, 23)_ = 1.961, p = 0.1636; (m) Ipsi, F_(2, 24)_ = 4.017, p = 0.0313, Contra, F_(2, 23)_ = 0.1066, p = 0.8993). PolyI:C injected into the left CeA significantly upregulated *Tlr3* and *Tlr4* expression on the ipsi- but not contra-lateral CeA and downregulated *Reln* on both sides, while LPS induced significant changes in *Tlr4* and *Reln* expression levels in the ipsi-, but not contra-, lateral CeA (Figures 8n–r, one-way ANOVA, (n) Ipsi, F_(2, 25)_ = 9.676, p = 0.0008, Contra, F_(2, 25)_ = 0.6930, p = 0.5094; (o) Ipsi, F_(2, 25)_ = 18.18, p < 0.0001, Contra, F_(2, 25)_ = 0.6393, p = 0.5361; (p) Ipsi, F_(2, 25)_ = 2.329, p = 0.1181, Contra, F_(2, 25)_ = 0.009821, p = 0.9902; (q) Ipsi, F_(2, 25)_ = 4.838, p = 0.0168, Contra, F_(2, 25)_ = 7.737, p = 0.0024; (r) Ipsi, F_(2, 25)_ = 0.4122, p = 0.6666, Contra, F_(2, 25)_ = 2.422, p = 0.1093). These results suggest that LPS or PolyI:C injection into either the left or right CeA can still induce changes in mRNA expression of neuroinflammatory markers at the 7-day time pint, when there is right-hemispheric lateralization of pain-like behaviors in females.

Taken together, these data showed that mRNA expression changes can be induced in both the left and right CeA ipsilaterally to the exogenous pharmacological injection, confirming the involvement of neuro-inflammatory mechanisms after LPS or PolyI:C application. However, these findings do not fully account for the lateralized effects (to the right CeA) observed in nociceptive behaviors over time (from 3 to 7 days post-injection). Overall, the lack of lateralized molecular changes of neuroimmune signaling suggests that the development of lateralization of neuroimmune signaling-induced pain-like behaviors from 3 to 7 days post-injection is not the direct consequence of the molecular changes but that perhaps protective mechanisms are engaged to inhibit the coupling to pain facilitation.

## 4 Discussion

In this research study, we sought to characterize the coupling of neuroimmune signaling to pain-like behaviors, focusing on sex, temporal, and hemispheric specific differences. The results of stereotaxic injections of PolyI:C or LPS into the CeA show that 1) exogenously-induced neuroimmune activation in the left and right CeA drives evoked nocifensive and affective behaviors in male and female rats 3 days post-induction (Figures 1, 3); 2) these effects became lateralized to the right CeA at the later (7 days) stage in female rats but not in male rats (Figures 2, 4); 3) no striking effects were observed on non-evoked anxiety-like behaviors regardless of the treatments (PolyI:C or LPS), hemispheres and sexes (Figures 5, 6); 4) changes in mRNA expression of pro-inflammatory markers were observed in the left and right CeA 3 days after the injection of neuroimmune activators (PolyI:C or LPS) and persisted 7 days after injection (Figures 7, 8).

The key conclusion is that both the left and right CeA are capable of undergoing neuroimmune activation and driving nocifensive and affective behaviors in males and females. This is different from the hemispheric laterization observed in pain models, perhaps suggesting an important role of the specific type of CeA activation induced by inputs such as from the PB. However, right-hemispheric lateralization of behavioral coupling, but not neuroimmune activation, developed over several days in females, but not in males, and was observed with the activation of TLR3 rather than TLR4. The right-hemispheric dominance observed in this study appears to depend on sex, treatment, time point, and behavioral outcome. This finding points to female-specific processes that uncouple the CeA activity from descending or ascending pain modulation on the output side.

Immune cells are involved in important physiological functions within the nervous system, including the regulation of homeostasis, inflammation, synaptogenesis and tissue repair. Dysregulations of the neuroimmune system influence nociceptive signaling and contribute to the development and maintenance of pain (Chen et al., 2023; Ji et al., 2018). TLRs are crucial elements responsible for the initiation of the innate immune response (Medzhitov, 2001). TLR4 recognizes and is activated by LPS, the major constituent of the outer membrane of Gram-negative bacteria. LPS has been extensively used in preclinical settings to investigate the role of TLR4 (Cavaillon, 2018; Zamyatina and Heine, 2020; da Silva et al., 2024). The activation of TLR3 by its selective agonist PolyI:C, a synthetic analogue of double-stranded RNA typically found in some viruses, produces a robust inflammatory response through the activation of the innate immune system (Harris et al., 2013). Previous studies reported increased pain-like behaviors following LPS administration. For example, systemic LPS induced mechanical and thermal hyperalgesia in neonatal (Hsieh et al., 2018) and adult (Yoon et al., 2012; Yang et al., 2019; Zhao et al., 2014) animals. Moreover, local injection of LPS into the plantar paw surface or the infra-orbital area enhanced mechanical pain-like responses in naïve rats (Cunha et al., 2000; Boucher et al., 2018). Importantly, evidence from preclinical studies showed that systemic LPS administration induced microglial and astrocytic activation in the spinal cord of neonatal (Hsieh et al., 2018) and adult (Yoon et al., 2012) rats, as well as in several brain areas, including hippocampus, cortex, hypothalamus, and amygdala (Oliveira-Lima et al., 2019; Yang et al., 2016), demonstrating the involvement of the innate immunity in the LPS-induced effects. Little is known about LPS actions in the brain. To the best of our knowledge no study has addressed the pain-related behavioral effects of LPS directly within the amygdala on either sex. Intracerebroventricular LPS caused microglia activation accompanied by an increase in the release of pro-inflammatory cytokines in the hippocampus (Zhao J. et al., 2019), and an augmented expression of microglial and astrocytic gene markers in the whole mouse brain (Aid et al., 2008). Bilateral injection of LPS into the CeA of male and female mice resulted in local increased microglia cell body size in both sexes (Dworsky-Fried et al., 2022), confirming microglia recruitment and activation. Our group previously linked enhanced levels of TLR4 in the amygdala to neuropathic pain (Santos et al., 2023), suggesting a critical contribution of this signaling pathway to endogenous pain mechanisms. The results of this study provide direct evidence for pronociceptive effects of LPS-induced TLR4 activation in the amygdala in naïve rats. It has been reported that systemic or intracerebroventricular LPS induced cognitive and memory deficits in naïve mice (Zhao J. et al., 2019; Ali et al., 2024).

Effects PolyI:C treatment in preclinical setting have been investigated. A recent study demonstrated that human microglia challenged (24 h) with PolyI:C showed reduced cell viability and increased release of pro-inflammatory cytokines (IL-18 and IL-12) and chemokines (CCL2 and CXCL-16) in the media. Systemic injection of PolyI:C induced cognitive impairments without affecting motor and neuromuscular function in mice, and these effects were associated with enhanced expression of the genes coding for TNF-α, IL-6 and IL-1β (Altahrawi et al., 2025). Interestingly, pro-inflammatory markers were enhanced in the hippocampus but not in the cortex, arguing for region-specific differences with respect to neuroinflammation processes in the brain. These behavioral findings were in line with a different study reporting the development of depression- and anxiety-like behaviors after recurring systemic exposure of PolyI:C in rats (Gibney et al., 2013). Our data showed a reduction of time spent in the center of the OFT but no significant effects were observed in the EPM 3 but not 7 days after intra-CeA PolyI:C in male animals, which is consistent with a reversible TLR3-mediated anxiogenic response. PolyI:C treatment had no effects in female rats, suggesting that TLR3 stimulation in the CeA does not drive anxiety-like behaviors in females. Direct evidence of pronociceptive effects of the exogenous activation of TLR3 in the brain is still lacking. In neuropathic pain conditions, increased TLR3 mRNA and protein expression was observed in the rat spinal cord (Chen and Lu, 2017) and knocking out TLR3 ameliorated the cognitive and sensory deficits (Zhang et al., 2023), suggesting a critical role of TLR3 signaling in neuropathic pain. Pharmacological strategies using intrathecal or systemic PolyI:C injection increased mechanical and thermal nociceptive responses in neuropathic (Chen and Lu, 2017) and sham (Zhang et al., 2023) rats, proving evidence for the facilitatory and pronociceptive effects of TLR3 activation signaling.

An important question addressed in our study was about lateralized behavioral effects of exogenous activation of neuroimmune signaling in the amygdala. The primary goal of the study was to examine the effects of neuroimmune activation on hemisphere-specific pain-related behaviors in males and females. As an initial step of investigating molecular mechanisms underlying these effects, we performed gene expression analysis of selected molecular targets in animals showing largest effects. We found that female, but not male, rats developed a right-hemispheric laterization, which could be due to a lack of neuroimmune activation in the left CeA or uncoupling of neuroimmune activation from behavioral consequences. To address this question, we performed qPCR analysis of the left and right CeA tissue in femals at 3 and 7 days and found changes in the mRNA expression of neuroinflammatory markers after LPS or PolyI:C administration consistent with the activation of inflammatory processes. We limited the qRT-PCR analyses to female animals because the behavioral effects were more pronounced in females, making this group the most suitable for exploring potential underlying molecular mechanisms. *Tlr3* and *Tlr4* play a central role in neuroinflammation and our results show their upregulation in the CeA after PolyI:C or LPS injection. *Tlr3* and *Tlr4* signal through myeloid differentiation factor 88 (MyD88)- and TIR-domain-containing adapter-inducing interferon-β (TRIF)-dependent pathways to induce robust production of pro-inflammatory cytokines (e.g., TNF-α, IL-1β) and chemokines, primarily from microglia and astrocytes (Alhamdan et al., 2024; Balan et al., 2024). The contribution of the neuroimmune system to neuronal excitability and synaptic plasticity in the brain remains an understudied area of research. TLR3-driven signaling has been shown to modulate glutamatergic transmission and contribute to persistent pain states, while pharmacological or genetic inhibition of TLR3 attenuates neuroinflammation and reduces pain-like behaviors in preclinical models (Grace et al., 2014; Bruno et al., 2018). Elevated *Ccl5* signaling through its receptors (e.g., CCR5) has been linked to increased synaptic excitability and facilitation of nociceptive transmission, contributing to both sensory and affective dimensions of pain (Gao and Ji, 2010; Garcia-Dominguez, 2025). Similarly, stimulation of TLR3 and TLR4 pathways enhances IL-23 signaling, which promotes expression of IL23R and production of IL-17, resulting in the sustainment of inflammatory cascades and maintenance of pain (Jiang et al., 2022). *Cxcl10* and *Cxcl1*2 promote neuroinflammation by the activation of their respective receptors *Cxcr3* and *Cxcr4* on neuroimmune elements, which modulate neuronal–glial interactions and facilitate excitatory transmission contributing to pain states (Chen et al., 2019; Liu et al., 2019; Chen et al., 2025). Our data show a reduction of *Olig1* levels, a factor involved in oligodendrocyte differentiation and myelin repair processes that can be disrupted during neuroinflammatory conditions (Zhao H. et al., 2019). Although the role of oligodendrocytes in the brain in pain is not clear and generally understudied except for demyelinating disorders, impaired oligodendrocytic functions and myelin dysregulation can contribute to maladaptive neural network activity and persistent pain states (Kim and Angulo, 2025; Presto et al., 2026). Reduced *Reln* signaling, which is important for synaptic plasticity, is linked to dysfunctional GABAergic transmission, resulting in a shift toward enhanced excitatory activity which is associated with the development of several neurological disorders (Joly-Amado et al., 2023; Markiewicz et al., 2023). *Bdnf* is a critical regulator of neuronal survival and synaptic plasticity within the central nervous system, and dysregulation of its signaling pathway has been linked to pain condition (Mazzitelli et al., 2025). In this study, we observed an upregulation of proinflammatory factors (*Cxcl10*, *Cxcl12, Ccl5 and Il23r*) and *Tlr3* and *Tlr4* (the targets of our pharmacological manipulations), and a downregulation of factors involved in neuronal plasticity (*Olig1*, *Reln* and *Bdnf*). The key message here is that at the mRNA level, neuroimmune signaling was activated in both the left and right CeA and therefore can not explain the right-CeA lateralization observed in the behavioral assays in female animals, suggesting the involvement of different underlying mechanisms to the pain-related right hemisphere dominance that may involve uncoupling from output/to ascending and descending systems.

This research project aimed to address if the well-documented right-hemispheric lateralization in preclinical pain models (Thompson and Neugebauer, 2017; Kolber et al., 2026; Ji and Neugebauer, 2009) is also observed with the exogenously induced amygdala neuroinflammation in the absence of extra-amygdala changes such as tissue injury or pain pathway activation. Lateralization observed with the exogenous activation of the neuroimmune system in the amygdala would provide evidence for intrinsic hemispheric differences underlying pain-related lateralization mechanisms. Our data show that female naïve animals developed right amygdala lateralization at 7 days after intra-CeA PolyI:C treatment, while no clear lateralization patterns emerged in the male groups. Both treatments (PolyI:C and LPS) were able to drive pain-like behaviors at the early stage (3 days post-injection). A previous study reported reduced density of parvalbumin interneurons and perineuronal nets in the basolateral amygdala (BLA) in the offspring of females rats treated with PolyI:C, which was more pronounced in the female offspring group (Casquero-Veiga et al., 2022), suggesting TLR3-related sex-differences within the amygdala. In TLR3-deficient mice, impairments in the amygdala-dependent memory and anxiety-behaviors were observed (Okun et al., 2010), pointing to a critical role of endogenous TLR3 in amygdala mechanisms. Males showed depressive-like and avoidance behaviors that lasted several days after systemic LPS injection, while females seemed to be more resilient to the LPS challenge (Millett et al., 2019; Patel et al., 2023), supporting different sensitivity to the exogenously-induced TLR4 activation between sexes.

The regulation and downstream consequences of TLR3-and TLR4-signaling and cell-type specificity remain to be explored in different conditions. Our findings demonstrate that exogenously induced neuroimmune signaling activation in the CeA drives pain-like behaviors in naïve female and male rats, but sex differences with respect to the right hemispheric lateralization of pain processing develop over time and can not be explained by intrinsic differences in neuroimmune signaling in the amygdala itself.

## Data Availability

The original contributions presented in the study are included in the article/Supplementary Material, further inquiries can be directed to the corresponding author.
