# Supplementary material for: Sex-specific lateralization of the effects of exogenously-induced neuroimmune activation in the amygdala on pain-like behaviors
Source: Front Pharmacol. 2026 Jul 17;17:1883574. doi: 10.3389/fphar.2026.1883574 (PMC13423922; doi:10.3389/fphar.2026.1883574)
Supplement: Supplementary file 1 [file Supplementaryfile1.docx]

Supplementary Material

1. **Methods**

For general information about emotional responses refer to section 2.4 in main text. Vocalizations in the audible (20 Hz to 16 kHz) and ultrasonic (25 ± 4 kHz) ranges were measured in female and male rats. A microphone connected to a preamplifier was used to record audible vocalizations, and a bat detector connected to a filter and amplifier measured ultrasonic vocalizations (UltraVox four-channel system; Noldus Information Technology). Vocalizations were evoked by brief (10 s) normally innocuous (300-500 g/6 mm2) stimuli applied to the contra- or ipsi-lateral (to the side of the injection) hind paws using a calibrated forceps. Vocalizations were recorded for 1 min.

## Supplementary Figure 1

**Supplementary Figure 1.** **Effects of LPS and Poly I:C injected into right or left CeA on the innocuous vocalizations in females and males.** In females at the 3 day-time point, LPS or Poly I:C injected into the right and left CeA had no effects on the audible vocalizations (**a & b**) evoked by the innocuous stimulation of the contra- and ipsi-lateral hind paws compared to vehicle. LPS, but not Poly I:C, injected into the left, but not right, CeA significantly increased the innocuous vocalizations in the ultrasonic range (**c & d**) compared to the vehicle treated group. No effects were observed after LPS or Poly I:C into the left or right CeA 7 days after injections (**e-h**). In males at the 3 day-time point, LPS or Poly I:C injected into the right and left CeA had no effects on the audible vocalizations (**i & j**) evoked by the innocuous stimulation of the contra- and ipsi-lateral hind paws compared to vehicle. LPS, but not Poly I:C, administered into the left, but not right, CeA enhanced the vocalizations in the ultrasonic range (**k & l**). 7 days after injection, LPS, but not Poly I:C into left and right CeA resulted in increased audible vocalizations evoked by the innocuous stimulation of ipsi-, but not contra-, lateral paws compared to the vehicle treated group (**m & n**), while no effects were observed on the ultrasonic vocalizations regardless of the treatment and hemispheres (**o & p**). Bar histograms show mean ± SEM. *, ** p < 0.05, 0.01 compared to vehicle, two-way ANOVA with Dunnett’s posthoc tests. (**a**) Contra vs Ipsi, F_(1, 32)_=0.07935, p=0.7800; Treatment, F_(2, 32)_=0.2873, p=0.7522; Interaction, F_(2, 32)_=0.2183, p=0.8051; (**b**) Contra vs Ipsi, F_(1, 36)_=0.5583, p=0.4598; Treatment, F_(2, 36)_=3.861, p=0.0303, Interaction, F_(2, 36)_=0.1812, p=0.8351; (**c**) Contra vs Ipsi, F_(1, 34)_=0.5633, p=0.4581; Treatment, F_(2, 34)_=2.475, p=0.0992; Interaction, F_(2, 34)_=0.8788, p=0.4245; (**d**) Contra vs Ipsi, F_(1, 36)_=0.1198, p=0.7313; Treatment, F_(2, 36)_=9.285, p=0.0006; Interaction, F_(2, 36)_=0.4169, p=0.6622; (**e**) Contra vs Ipsi, F_(1, 48)_=0.07326, p=0.7873; Treatment, F_(2, 48)_=0.7145, p=0.4946; Interaction, F_(2, 48)_=0.08743, p=0.9164; (**f**) Contra vs Ipsi, F_(1, 47)_=0.9390, p=0.3375; Treatment, F_(2, 47)_=1.585, p=0.2156; Interaction, F_(2, 47)_=2.264, p=0.1151; (**g**) Contra vs Ipsi, F_(1, 47)_=0.1128, p=0.7385; Treatment, F_(2, 47)_=0.2438, p=0.7846; Interaction, F_(2, 47)_=0.07401, p=0.9288; (**h**) Contra vs Ipsi, F_(1, 49_=0.4679, p=0.4972; Treatment, F_(2, 49)_=0.08231, p=0.9211; Interaction, F_(2, 49_=0.7446, p=0.4808; (**i**) Contra vs Ipsi, F_(1, 36)_=0.2672, p=0.6084; Treatment, F_(2, 36)_=0.1245, p=0.8833; Interaction, F_(2, 36)_=0.5136, p=0.6027; (**j**) Contra vs Ipsi, F_(1, 35)_=0.03056, p=0.8622; Treatment, F_(2, 35)_=3.793, p=0.0323; Interaction, F_(2, 35)_=0.2149 , p=0.8077; (**k**) Contra vs Ipsi, F_(1, 35)_=1.294, p=0.2630; Treatment, F_(2, 35)_=0.03708, p=0.9636; Interaction, F_(2, 35)_=0.6942, p=0.5062; (**l**) Contra vs Ipsi, F_(1, 34)_=1.621, p=0.2115; Treatment, F_(2, 34)_=4.441, p=0.0193; Interaction, F_(2, 34)_=4.649, p=0.0164; (**m**) Contra vs Ipsi, F_(1, 39)_=0.3394, p=0.5635; Treatment, F_(2, 39)_=5.518, p=0.0078, Interaction, F_(2, 39)_=0.4203, p=0.6598; (**n**) Contra vs Ipsi, F_(1, 35)_=0.09664, p=0.7577; Treatment, F_(2, 35)_=0.5279, p=0.5945; Interaction, F_(2, 35)_=5.060, p=0.0117; (**o**) Contra vs Ipsi, F_(1, 40)_=0.2930, p=0.5913; Treatment, F_(2, 40)_=4.579, p=0.0162; Interaction, F_(2, 40)_=0.04289, p=0.9581;(**p**) Contra vs Ipsi, F_(1, 36)_=1.369, p=0.2497; Treatment, F_(2, 36)_=0.4568, p=0.6369; Interaction, F_(2, 36)_=0.08312, p=0.9204.
